# Contributions of T cell dysfunction to the resistance against anti-PD-1 therapy in oral carcinogenesis

**DOI:** 10.1186/s13046-019-1185-0

**Published:** 2019-07-10

**Authors:** Liling Wen, Huanzi Lu, Qiusheng Li, Qunxing Li, Shuqiong Wen, Dikan Wang, Xi Wang, Juan Fang, Jun Cui, Bin Cheng, Zhi Wang

**Affiliations:** 1 Guangdong Provincial Key Laboratory of Stomatology, Guanghua School of Stomatology, Stomatological Hospital, Sun Yat-Sen University, No.56, Lingyuan West Road, Yuexiu District, Guangzhou, 510055 Guangdong People’s Republic of China; 2 Key Laboratory of Gene Engineering of the Ministry of Education, State Key Laboratory of Biocontrol, School of Life Sciences, Sun Yat-Sen University, No. 135, Xingang West Road, Haizhu District, Guangzhou, 510275 Guangdong People’s Republic of China

**Keywords:** Oral precancerous lesion (OPL), Programmed cell death 1(PD-1), Central memory T cell (Tcm), Regulatory T cell (Treg), T cell immunoglobulin and mucin domain-containing protein 3 (TIM-3)

## Abstract

**Background:**

Programmed death 1 (PD-1) blockade has great effect in the prevention of oral precancerous lesions, but the drug resistance has also been observed. The determinants of immune resistance during the malignant transformation are poorly understood.

**Methods:**

Anti-PD-1 antibody was administered in the 4NQO-induced carcinogenesis mouse models. The mice were then subdivided into PD-1 resistance(PD-1R) group and PD-1 sensitive(PD-1S) group according to the efficacy. The expression of PD-1 and PD-L1, and the abundance of CD3^+^ T cells in tumor microenvironment between the two groups was tested by immunohistochemistry. In addition, the activation and effector functions, as well as the accumulation of immunosuppressive cells and expression of immune checkpoints of T cells in the draining lymph nodes and spleen between PD-1R and PD-1S group were analyzed by flow cytometry.

**Results:**

Our results showed that T cell infiltration in tumor microenvironment, effector T cell cytokine secretion and central memory T cell accumulation in peripheral lymphoid organs were all inhibited in the anti-PD-1 resistance group. Furthermore, we found that an increase of regulatory T cell (Treg) population contributed to the resistance of the anti-PD-1 therapy. Notably, TIM-3 was found to be the only immunosuppressive molecule that mediated the resistance to anti-PD-1 therapy in the oral malignant transformation model.

**Conclusions:**

Our findings identified a novel mechanism that T cell dysfunction contributes to the immune resistance during the malignant transformation of the oral mucosa. This study provides new targets for improving the efficacy of immunotherapy for early stage of tumorigenesis.

**Electronic supplementary material:**

The online version of this article (10.1186/s13046-019-1185-0) contains supplementary material, which is available to authorized users.

## Background

Cancer immunotherapy has become a promising approach in recent years, and the blockade of immune checkpoints, such as programmed death receptor 1 (PD-1) or programmed death ligand 1 (PD-L1), has been an attractive therapeutic method [1–3]. The engagement of PD-1 by PD-L1 will cause T cell exhaustion, the state in which the antitumor functions of T cells are greatly inhibited [4]. The blockade of PD-1 or PD-L1 will relieve T cell immunosuppression in the tumor microenvironment and further inhibit tumor growth [5]. Correspondingly, in an effort to explore the potential role of PD-1 in the initiation of oral carcinogenesis, our previous study also demonstrated that blockade of the PD-1/PD-L1 pathway can effectively inhibit the malignant transformation of the oral mucosa in vivo [6], and this blockade showed an encouraging degree of efficacy in the prevention of oral precancerous lesions (OPLs).

However, primary resistance to anti-PD-1 therapy is still observed in many kinds of tumors, resulting in unsatisfactory response rates and poor prognosis. For example, the response rate was reported to be lower than 35% in advanced malignant melanoma patients administered anti-PD-1 antibodies [7], and in non-small cell lung carcinoma patients, the positive response rate was only 20% [8].

Here, we employed the 4-nitroquinoline-1-oxide (4NQO)-induced carcinogenesis model in immunocompetent C57BL/6 mice and administered anti-PD-1 antagonistic antibodies to the mice. Our study revealed that a small group of mice failed to respond to the anti-PD-1 antibody treatment, leading to progression into carcinoma in situ or invasive carcinoma. However, to date, little is known about the mechanism of drug resistance to anti-PD-1 therapy in the context of malignant transformation in oral premalignant lesions. In present study, we found T cell dysfunction contributes to the immune resistance during the malignant transformation of the oral mucosa. This study provides new targets for improving the efficacy of immunotherapy for early stage of tumorigenesis.

## Methods

### Mice

Six-week-old female C57BL/6 mice (*n* = 28) were purchased from Guangzhou University of Chinese Medicine. All mice were maintained in a specific pathogen-free facility, and experimental procedures were conducted under institutional guidelines that comply with national laws and policies. The study protocols were approved and performed in accordance with the guidelines of the Institutional Animal Care and Use Committee of Sun Yat-Sen University.

### 4NQO-induced oral tumorigenesis model

The carcinogen 4NQO (Sigma-Aldrich) was dissolved in propylene glycol (Sigma-Aldrich) at 4 mg/ml to create a stock solution, which was stored at 4 °C and diluted in autoclaved water to a final concentration of 50 μg/ml. For the malignant transformation of the oral mucosa model, 6-week-old female C57BL/6 mice were exposed to 4NQO in the drinking water for 16 weeks, and the water was replaced once a week. After the 16-week carcinogen treatment, the drinking water was switched to distilled water. The mice were analyzed for oral lesions and weighed at different times for up to 16 or 20 weeks.

### Antibody treatment

**Fig. 1:**
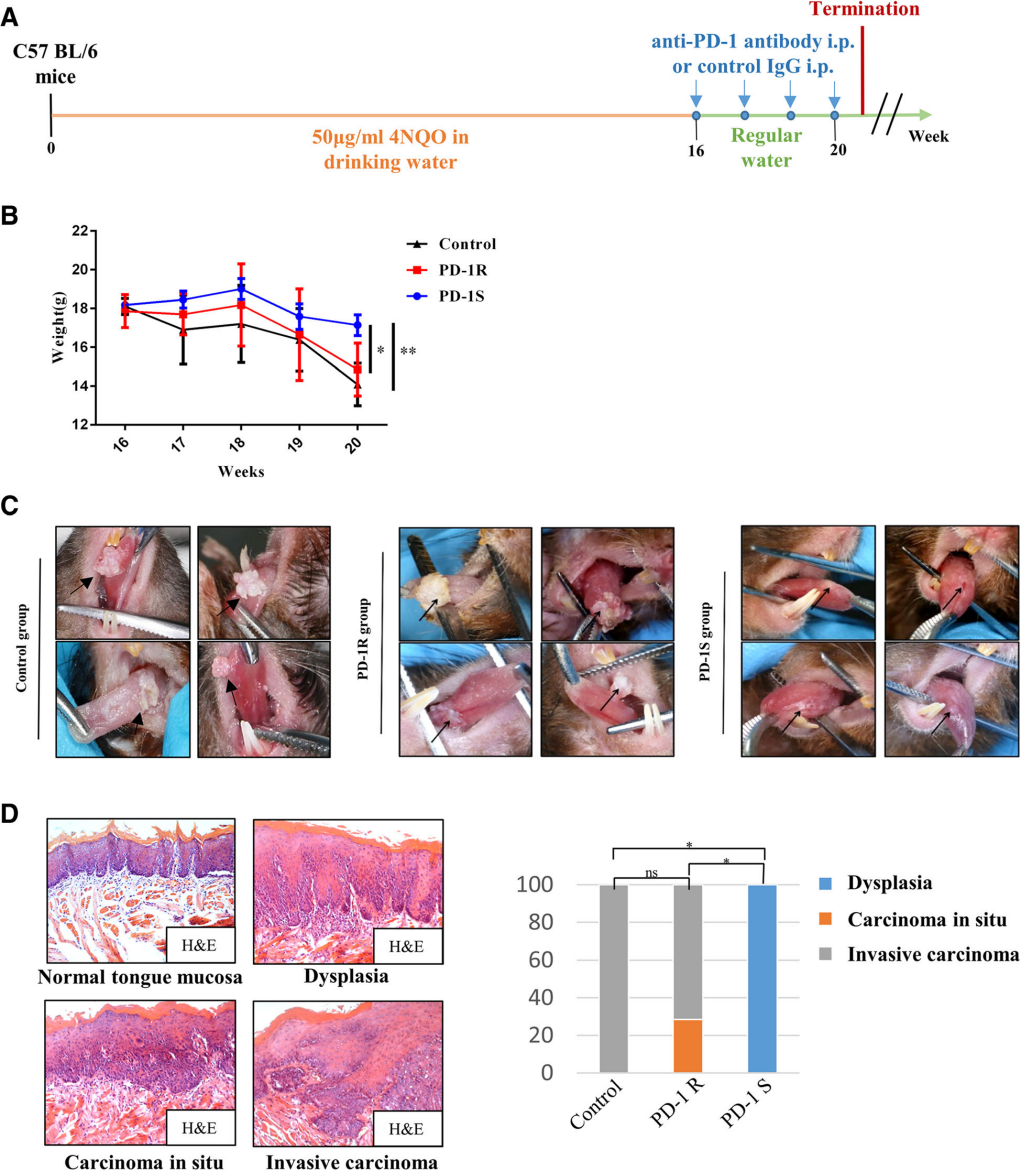
PD-1 blockade resistance occurred in the oral malignant transformation mouse model. **a** The schematic picture shows the 4NQO treatment and anti-PD-1 antibody(*n* = 23) and control IgG (vehicle control, *n* = 5) drug delivery strategies in C57BL/6 mice. **b** Body weight (g) was measured and documented for the control group and anti-PD-1 group (the PD-1R and PD-1S groups) once a week. Significant weight loss was observed in the PD-1R group at week 20. The data are presented as the mean ± SEM (one-way repeated-measures ANOVA, *P < 0.05, **P < 0.01). **c** Representative macroscopic observation of the lingual mucosal lesions after treatment with control IgG(left panel) or anti-PD-1 antibody in the PD-1R group (middle panel) and PD-1S group (right panel). For PD-1R group, similarly with control group, leukoplakia-like lesions with smooth surfaces progressed into white masses with cauliflower-like (upper left), rough and granular (upper right) or exogenous verrucous surfaces (lower right and left). The lingual mucosal lesions treated with anti-PD-1 antibodies maintained a wrinkled paper-like appearance macroscopically in PD-1S group. **d** Representative hematoxylin and eosin (H&E) staining of dysplasia, carcinoma in situ (pre-invasive carcinoma) and invasive carcinoma. Statistical significance was determined by the Kruskal-Wallis test, *P < 0.05

Anti–mouse PD-1 monoclonal antibody (mAb; clone G4) were kindly provided by Lieping Chen (Yale University School of Medicine, New Haven, CT, USA). Control IgG was used as a negative control for the tumorigenesis experiments. According to the differences in their tongue mucosal lesions, the mice were randomly divided into control group (control IgG, 200 μg, intraperitoneal, weekly; *n* = 5 mice) and an anti-PD-1 group (anti-PD-1 antibody, 200 μg, intraperitoneal, weekly; *n* = 23 mice) at 16 weeks after the oral gavage with 4NQO. Control IgG or the anti-PD-1 antibody was administered for four consecutive weeks. The mice were humanely euthanized at the end point (20 weeks). The timing of these treatments is shown graphically in Fig. 1a.

### Histology and pathological analysis

Oral lesions were identified and photographed from week 16 to week 20 once a week.

The mice were euthanized after treatment. Harvested oral lesions were fixed in 10% formalin, paraffin embedded and sectioned into 4-μm sections. Hematoxylin and eosin (H&E) staining was performed on the tongue sections. For routine histological analysis, the histopathological grading (Dysplasia—Mild/Moderate Dysplasia, Carcinoma in situ—Severe Dysplasia, Invasive Carcinoma) was performed with a light microscope (Olympus Optical) and reviewed by 2 certified pathologists. Images of the tongue tissue sections were acquired at 200× magnification.

### Immunohistochemistry

Immunohistochemistry was performed on deparaffinized sections with rabbit monoclonal anti-mouse PD-1 (D7D5W; CST), rabbit monoclonal anti-mouse PD-L1 (D5V3B; CST), rabbit monoclonal anti-mouse CD3 (Clone 17A2; R&D Systems), rabbit monoclonal anti-mouse TIM-3(D3M9R; CST), and rabbit monoclonal anti-mouse Foxp3(D6O8R; CST) antibodies. The immunostaining was visualized with the DAB Detection Kit (Gene Tech, China) using a peroxidase and diaminobenzidine substrate. The sections were counterstained with Mayer’s hematoxylin, examined by using a light microscope (Olympus Optical) and reviewed by 2 certified pathologists. Images of the tumor tissue sections were acquired at 100×, 200× and 400× magnifications.

The positive cells were counted under 400× magnification, and five randomly selected independent microscopic fields were counted for each sample to ensure that the data were representative and homogeneous. The immunohistochemical analysis were blindly scored by two certified pathologists. The expression of PD-L1, PD-1, TIM-3 and Foxp3 on tumor infiltrating immune cells was scored according to criteria described in ref. [9]. Specimens were given a score as follows: 1,< 5%; 2, 5–< 10%; 3, ≥10%. The CD3+ cells were quantified by the percentage of total number of cells according to the criteria described in ref. [10] and was slightly modified. The score was listed as follows: 1,< 10%; 2, 10–20%; 3, > 30%.

### Flow cytometry

A single cell suspension was prepared from the spleen and draining lymph nodes of the mice. Immune cells were stained with anti-mouse antibodies against CD3, CD4, CD8, CD11b, Gr-1, CD44, CD62L, PD-1, TIM-3, CTLA-4, and LAG-3 at 4 °C for 30 min. All antibodies were purchased from eBioscience. For the intracellular staining for IL-2, IFN-γ, and TNF-α, the cells were stimulated with PMA (eBioscience) and ionomycin (eBioscience) for 5 h at 37 °C with 5% CO2. GolgiPlug (BD) was added at a dilution of 1:200 after the first hour of the incubation. For the intracellular cytokine staining, the cells were washed, stained with surface marker antibodies, fixed and permeabilized with fixation/permeabilization and permeabilization buffer (eBioscience) and intracellularly stained with anti-IL-2, anti-TNF-α, and anti-IFN-γ antibodies according to a standardized protocol. Staining for intracellular Foxp3, a regulatory T cell (Treg) marker, involved staining with surface marker antibodies, fixing, permeabilizing, and staining the cells with an anti-Foxp3 antibody for 30 min at room temperature (eBioscience). Samples were analyzed on a BD FACSVerse flow cytometer and analyzed with FlowJo software version 10.

### Statistical analysis

Measurements are expressed as the mean ± standard error of mean (SEM). Statistical analysis of the differences in animal weight was performed using one-way repeated-measures ANOVA. Kruskal-Wallis test was used to compare the means across the 3 groups. Student’s t test was used to compare between 2 groups. FACS results were analyzed with FlowJo software version 10. All statistical analyses were performed with GraphPad Prism version 7.0, which was also used to create all graphs. *P* values less than 0.05 was considered statistically significant. *P < 0.05, **P < 0.01, ***P < 0.001.

## Results

### Drug resistance was associated with persistent malignant transformation in oral precancerous lesions

To distinguish the drug-resistant mice from the sensitive mice, we first established the 4NQO-induced carcinogenesis model in immunocompetent C57BL/6 mice and administered an anti-PD-1 monoclonal antibody (mAb) to the anti-PD-1 group and control IgG to the control group once per week for 4 consecutive weeks, as previously described (Fig. 1a) [6]. During the course of treatment, we observed that a subgroup of anti-PD-1-treated mice suffered significant weight loss, which was similar to that of the control group (Fig. 1b). In addition, similarly with control group, the leukoplakic lesions in this subgroup progressed into white masses with a cauliflower-like or verrucous appearance by gross morphology (30.43%, 7/23 mice) (Fig. 1c). As shown in Fig. 1d, these lesions were classified microscopically as carcinoma in situ (28.58%, 2/7 mice) or even invasive carcinoma (71.42%, 5/7 mice) through H&E staining. Based on these characteristics, these mice were classified as the PD-1 resistance group (PD-1R group). In contrast, the rest of the mice in the anti-PD-1 group responded well to the anti-PD-1 antibodies (69.57%, 16/23 mice), and this response manifested as a relatively slow reduction in body weight (Fig. 1b) and no changes in the wrinkled paper-like lesion appearance and corresponded with hyperplasia (Fig. 1c) by histopathology (Fig. 1d); this subset of mice was then classified as the PD-1 sensitive group (PD-1S group).

### Resistance to PD-1 treatment altered the tumor immune microenvironment of the oral precancerous lesions

**Fig. 2:**
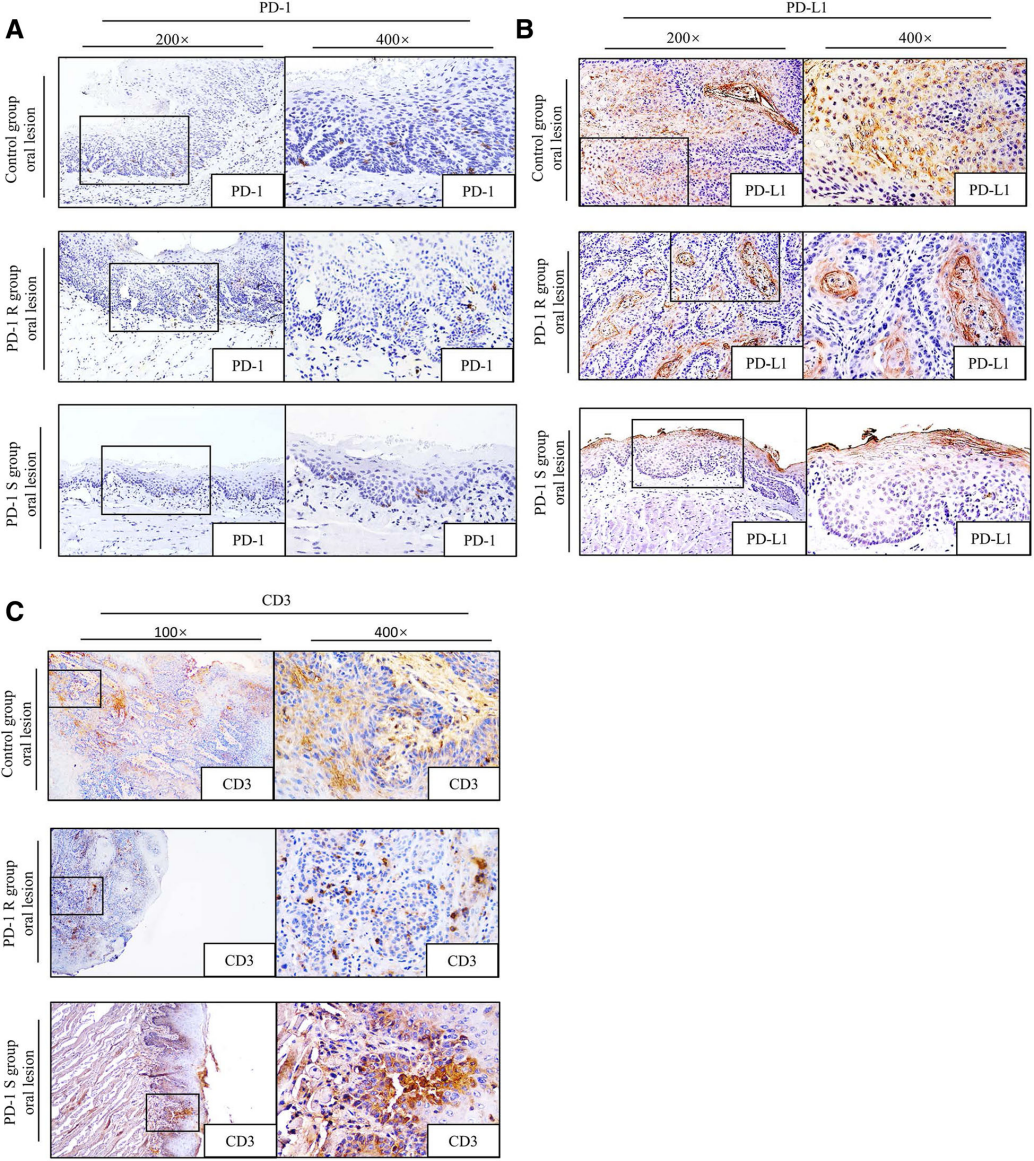
Expression levels of PD-1, PD-L1 and CD3^+^ T cell infiltration in PD-1R, PD-1S and control IgG groups. Representative immunohistochemically stained images of the tissue sections from the tongue. PD-1 (**a**) and PD-L1 (**b**) staining was detected in three groups. The expression of PD-1 and PD-L1 in the PD-1S and PD-1R groups were both lower than that in the control group, and the expression of PD-L1 in PD-1S group is significantly lower than PD-1R group(*P* < 0.05). **c** CD3^+^ T cell infiltration increased in the PD-1S group but decreased in the PD-1R group compared with the control group(*P* < 0.05). The boxes outlined with solid lines are the representative sites in the 100× or 200× pictures. The 400× images show high-magnification views of the boxed areas

Next, to determine whether the immune microenvironment was altered in the PD-1R group, immunohistochemical staining of PD-1, PD-L1 and CD3 were performed on tissue samples from the PD-1R, PD-1S and control IgG groups. Our previous study has shown PD-1 antibody treatment suppressed PD-1 expression on TILs and T cells in peripheral lymph tissues [6]. Consistently, as shown in Fig. 2a, the expression of PD-1 in the PD-1S and PD-1R groups were both lower than that in the control group, but the difference between the PD-1R and PD-1S group was no significance. However, as shown in Fig. 2b, the expression of PD-L1 in the PD-1R and PD-1S groups were both lower than that in the control group, and the expression of PD-L1 in PD-1S group is significantly lower than PD-1R group(Additional file 1: Table S1, *P* < 0.05). In addition, tumor-infiltrating CD3^+^ T cells were more accumulated in the PD-1S group than in the PD-1R and control group (Fig. 2c, Additional file 1: Table S1, *P* < 0.05). These findings revealed that insufficient CD3^+^ T cell infiltration might have contributed to the impaired antitumor immunity, resulting in drug resistance to the anti-PD-1 treatment.

### Drug resistance was associated with inhibition of central memory T cell accumulation and T cell effector functions

**Fig. 3:**
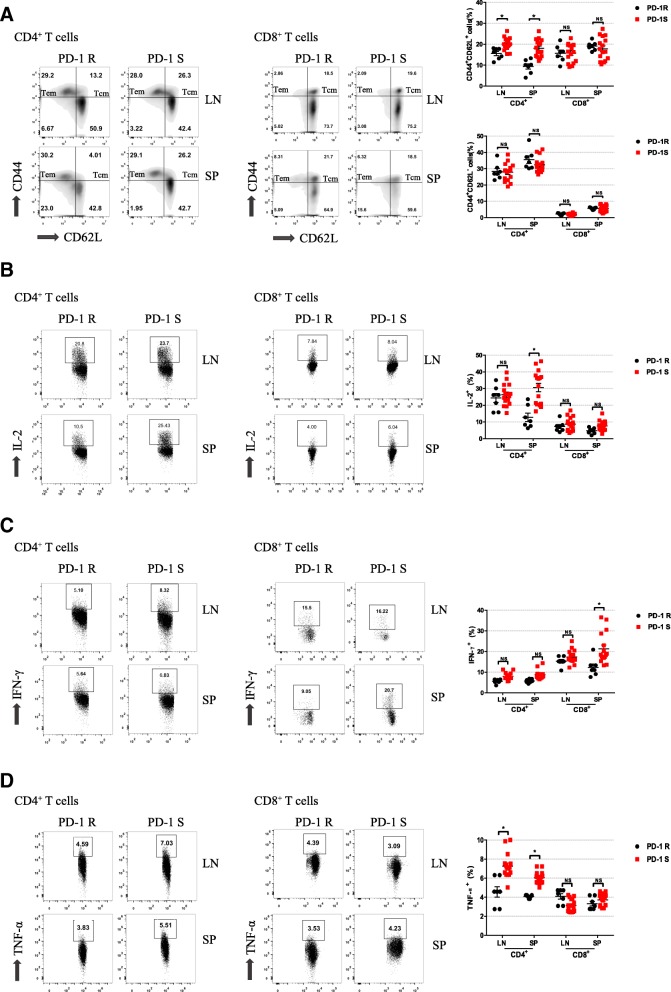
PD-1R mice exhibited decreased T cell accumulation and effector function. **a** Tcm and Tem cell populations in the LN and SP of the PD-1S (*n* = 16) and PD-1R (*n* = 7)groups were quantified. Representative flow cytometry plots show a decreased Tcm cell population in the PD-1R group. **b**, **c**, **d** Representative flow cytometric analysis and the percentages of CD4^+^ and CD8^+^ T cells expressing IL-2, IFN-γ, and TNF-α in the LN and SP of the PD-1 R and PD-1 S groups are shown. We found that the IL-2 and IFN-γ expression in the splenic CD4^+^ T cells and CD8^+^ T cells in the PD-1R group was significantly reduced compared to that in the PD-1S group. All data represent the mean ± SEM. Statistical significance was determined by Student’s t test, *P < 0.05, **P < 0.01, ***P < 0.001. LN, lymph node; SP, spleen

To further identify whether the activation of T cells was inhibited in the PD-1R group, the percentages of central memory T (Tcm, CD44^+^CD62L^+^) cells and effector memory T (Tem, CD44^+^CD62L^−^) cells was analyzed by flow cytometry. As shown in Fig. 3a, the percentages of CD4^+^ Tcm cells in the draining lymph nodes and spleen in the PD-1R group were significantly lower than those in the PD-1S group (*P* < 0.05); in contrast, no significant differences were observed between the two groups in regard to the percentages of CD8^+^ Tcm cells in either the lymph nodes or spleen. Moreover, neither the CD4^+^ nor CD8^+^ Tem cell populations showed significant differences in the peripheral lymphoid tissue (Fig. 3a), suggesting that the resistance to anti-PD-1 antibodies could be more attributed to the decrease in Tcm cells in oral malignant transformation. Furthermore, we examined the effector functions of the T cells by analyzing the production of IL-2, IFN-γ, and TNF-α. We found that the IL-2 and IFN-γ expression in the splenic CD4^+^ T cells and CD8^+^ T cells in the PD-1R group was significantly reduced compared to that in the PD-1S group. Additionally, the amounts of IL-2 and IFN-γ produced in the CD4^+^ T cells and CD8^+^ T cells in the draining lymph nodes, IL-2 in the CD8^+^ T cells in the spleen and IFN-γ in the CD4^+^ T cells in the spleen in the PD-1R group were lower than those produced by the same cell populations in the PD-1S group, although the differences were not statistically significant (Fig. 3b, c). The percentages of TNF-α in the CD4^+^ cells in the draining lymph nodes and spleen in the PD-1R group were significantly lower than those in the PD-1S group (*P* < 0.05) (Fig. 3d). These results indicated that the antitumor functions of the T cells in the PD-1R group were weakened, resulting in an “anergic” state.

### Increasing regulatory T cell infiltration was associated with drug resistance to anti-PD-1 therapy

**Fig. 4:**
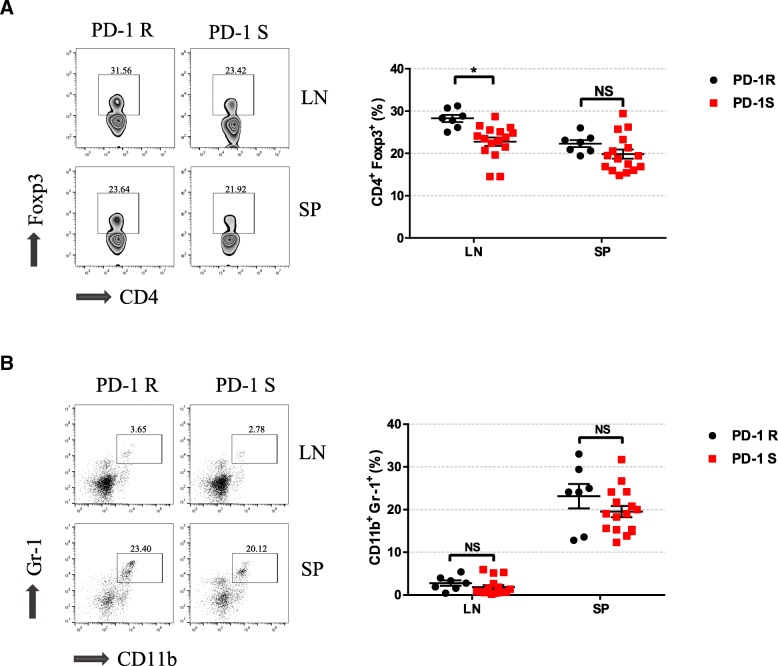
Relative distributions of key immunosuppressive cells after the anti-PD-1 antibody treatment. **a**, **b** Flow cytometry analysis was performed to characterize and quantify Tregs (CD4^+^Foxp3^+^) and MDSCs (CD11b^+^Gr-1^+^). Compared to the PD-1S group, the PD-1R group exhibited an increase in Treg accumulation. All data represent the mean ± SEM. Statistical significance was determined by Student’s t test, *P < 0.05. Tregs, regulatory T cells; MDSCs, myeloid-derived suppressor cells

It has been reported that some immunosuppressive cells, including Tregs and myeloid-derived suppressor cells (MDSCs), can exert immunosuppressive effects in the tumor microenvironment. Therefore, to determine whether these immunosuppressive cells are involved in resistance to PD-1 treatment, we detected the numbers of CD4^+^Foxp3^+^ Tregs and Gr1^+^CD11b^+^ MDSCs in the spleen and draining lymph nodes by flow cytometry. We noted a marked accumulation of Tregs in the lymph nodes of the PD-1R group compared to those of the PD-1S group (*P <* 0.05) (Fig. 4a). In addition, there were no differences between the two groups in the number of MDSCs in the spleen and lymph nodes (Fig. 4b). We further validated in the immunohistochemistry that Foxp3^+^ Tregs were accumulated in the immune microenvironment of PD-1R group(*P* < 0.05, Additional file 2: Figure S1A, Additional file 1: Table S2). Our findings indicated that Tregs, rather than MDSCs, might have contributed to the drug resistance to the anti-PD-1 antibodies.

### TIM-3 was involved in the resistance to anti-PD-1 treatment

**Fig. 5:**
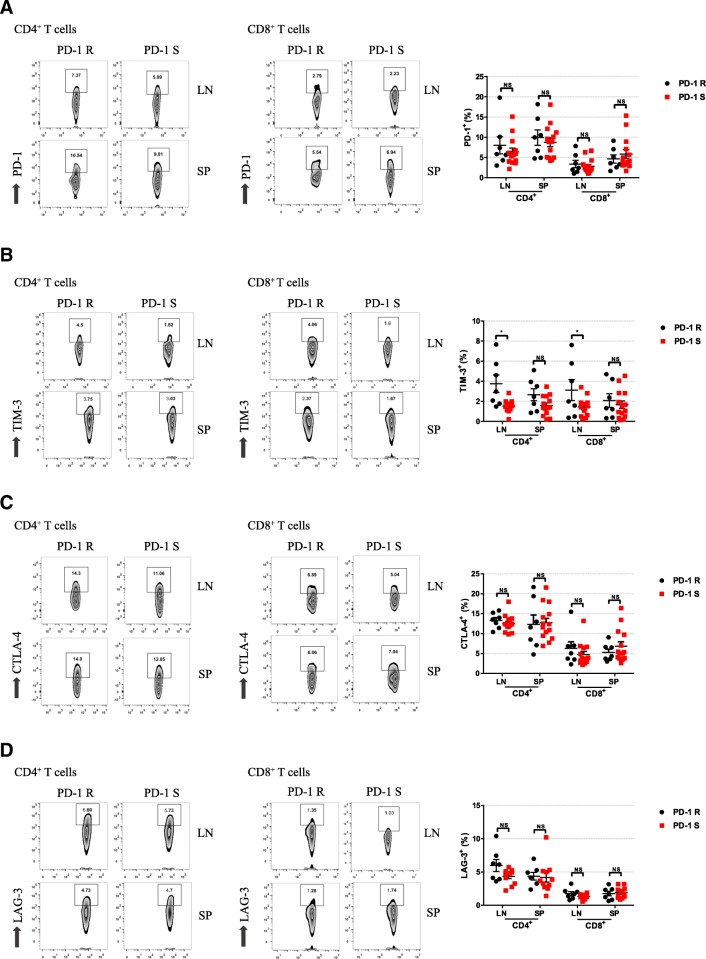
Higher frequencies of TIM-3-expressing T cells were observed in the PD-1R group. The expression of checkpoint inhibitors on CD4^+^ and CD8^+^ T cells was analyzed by flow cytometry for PD-1S group and PD-1R group. Representative flow cytometry dot plots show the analysis of checkpoint inhibitors expression on CD4^+^ and CD8^+^ T cells. The frequencies of PD-1^+^(**a**), TIM-3^+^(**b**), CTLA-4^+^(**c**) and LAG-3^+^(**d**) cells are shown. The data show that TIM-3 expression was significantly increased in the CD4^+^ and CD8^+^ cells in the LN and SP of the PD-1R group compared with PD-1 S group. All data represent the average ± SEM. Statistical significance was determined by Student’s t test, *P < 0.05, **P < 0.01

To further explore the potential molecular mechanism of drug resistance to PD-1 blockade in oral mucosal malignant transformation, the expression of the coinhibitory molecules PD-1, TIM-3, CTLA-4, and LAG-3 on CD4^+^ T cells and CD8^+^ T cells in the spleen and draining lymph nodes was analyzed by flow cytometry. Our results revealed that there was no difference of PD-1 expression between the PD-1R and PD-1S groups (Fig. 5a). In addition, TIM-3 was more highly expressed on the CD4^+^ T cells and CD8^+^ T cells in the draining lymph nodes of the PD-1R group than in the PD-1S group (*P <* 0.05); we also observed a modest increase in TIM-3 expression that did not achieve statistical significance in the CD4^+^ T cells and CD8^+^ T cells in the spleen (Fig. 5b). In addition, the expression of CTLA-4 and LAG-3 on the CD4^+^ T and CD8^+^ T cells in the spleen and lymph nodes was not significantly different between the groups (Fig. 5c, d). Interestingly, when subdivided into Tem and Tcm populations, we also found a relatively higher expression of TIM-3 on CD4^+^/CD8^+^ Tcm cells and CD8^+^ Tem cells in the lymph nodes of PD-1R group than that in PD-1S group(*P* < 0.05, Additional file 3: Figure S2A, B), whereas no significant differences were observed in spleen between the two groups(Additional file 3: Figure S2A, B). Furthermore, the TIM-3 expression was also significantly upregulated in tumor microenvironment of PD-1R group, which was confirmed by immunohistochemistry (*P* < 0.05, Additional file 2: Figure S1B, Additional file 1: Table S2). Our findings suggested that TIM-3 might be the key co-inhibitory molecule that mediates the drug resistance of the oral precancerous lesions to anti-PD-1 therapy.

## Discussion

In our previous study, we found that PD-1 blockade therapy can effectively prevent the formation of precancerous and/or cancerous lesions in the oral mucosa in vivo [6]. Nonetheless, some precancerous lesions exhibited poor responses to anti-PD-1 antibodies and progressed into cancer, which implies the existence of a potential drug resistance mechanism. Therefore, this study aimed to further explore the mechanism underlying drug resistance to anti-PD-1 therapy in the early course of malignant transformation in the oral mucosa. We discovered that the insufficient accumulation, activation and effector function of T cells were associated with poor response to anti-PD-1 treatment. Furthermore, Tregs and TIM-3 were found to be the possible cellular and molecular regulators, respectively, mediating the drug resistance against the anti-PD-1 therapy.

It is widely accepted that cancer immunotherapy, including strategies that lead to the persistence of effective T cell memory, is able to prevent cancer relapse and metastasis [11]. Tcm cells, a subset of memory T cells, are believed to confer more potent and durable antitumor immune responses in vivo than Tem cells [12], which implies that activating Tcm cells may be a promising antitumor approach. Recently, several studies have discovered that PD-1 blockade therapy not only reactivates effector T cells but also promotes the proliferation of Tcm cells, improving antitumor immunity [11, 13]. However, the relationship between drug resistance to anti-PD-1 antibodies and the population of Tcm cells in oral epithelial malignant transformation remains unknown. Our results, consistent with those of other reports, demonstrated that Tcm cells were significantly associated with resistance to anti-PD-1 therapy. Given that malignant transformation is a long-term process, we reason that the absence of Tcm cells might result in the failure to maintain durable immunity during anti-PD-1 treatment. These findings provide another possible way to enhance anti-PD-1 therapy, but the mechanism of memory T cell reinvigoration needs further exploration.

Furthermore, in an effort to investigate the immunosuppressive factors that produce resistance against anti-PD-1 antibodies in oral carcinogenesis, we found that Tregs and TIM-3 were potential candidates at the cellular and molecular levels, respectively. Tregs are one of the T cell subsets that regulate immune tolerance [14]. The accumulation of Tregs has been observed in the regional lymph nodes of mice with 4NQO carcinogen-induced premalignant oral lesions [15], and Treg levels continuously increase as lesions progress into oral cancer [16]. Moreover, Tregs are also reported to be associated with the resistance to anti-PD-1 therapy [17]. Our results suggested that Tregs may be involved in mediating resistance to anti-PD-1 antibodies in oral precancerous lesions. However, the exact mechanism is far from well understood. One possible explanation for the immunosuppressive mechanism is that high-affinity IL-2 receptors are constitutively expressed on Tregs, which allows Tregs to continuously absorb the IL-2 produced by effector T cells and in turn repress the activation and proliferation of effector T cells [18]. Thus, given previous results, further studies are needed to focus on the interaction between Tregs and effector or memory T cells.

It is known that apart from PD-1, there are a variety of coinhibitory receptors that negatively modulate T cell activation, including CTLA-4, LAG-3 and TIM-3 [19]. The existence of these immune checkpoints is partially associated with the low response rates to anti-PD-1 therapy in various kinds of cancer [20, 21]. Our results showed that among the immunosuppressive molecules, only the expression of TIM-3 on CD4^+^ T cells and CD8^+^ T were significantly upregulated in the peripheral lymphoid tissue in the PD-1R group, implying that TIM-3 is the potential key regulator of drug resistance to anti-PD-1 treatment. TIM-3, a negative immunomodulatory molecule originally discovered in 2002 [22], has become a new molecule of interest in immunotherapy. It is selectively expressed on T cells that secrete IFN-γ, including CD4^+^ T helper 1 (Th1) and cytotoxic CD8^+^ T cells. In addition, TIM-3 was recently found to be expressed on Tregs [23], as well as dendritic cells (DCs), natural killer cells (NKs), and macrophages [24]. Unlike PD-1, which has only one pair of ligands (PD-L1/2), TIM-3 has multiple ligands, including Galectin-9, HMGB1 and CEACAM1 [25–27]. The interactions between TIM-3 and its ligands transmit inhibitory signals, leading to T cell exhaustion and tumor cell immune escape [28, 29]. Moreover, lung cancer patients who developed adaptive resistance to anti-PD-1 treatment showed an increase in TIM-3 expression [21]. Furthermore, TIM-3 is also expressed by intratumoral Foxp3^+^ Tregs, which exhibit higher immunosuppressive activity than Foxp3- Tregs [23]. In addition, combination treatment with anti-PD-1 and anti-TIM-3 antibodies can effectively reverse T cell exhaustion and restore antitumor immunity [30]. Thus, our findings reveal a new approach to overcome PD-1 therapy resistance in oral precancerous lesions. Further studies are required to confirm the efficacy of combination therapy and explore the in-depth mechanisms of immune resistance.

## Conclusions

In all, the resistance of oral precancerous lesions to anti-PD-1 therapy was associated with the insufficient central memory T cell accumulation and reduction of T cell effector cytokine secretion. Notably, the up-regulation of Tregs infiltration and TIM-3 expression may contribute to drug resistance, and this finding provides promising targets to prevent oral precancerous lesions from undergoing malignant transformation.

## Additional files

Additional file 1: **Table S1.** Immunohistochemical analysis of the expression of PD-1, PD-L1 and CD3 in oral lesions. **Table S2.** Immunohistochemical analysis of the expression of Foxp3, TIM-3 in oral lesions. (DOCX 15 kb)

Additional file 2: **Figure S1.** Expression of Foxp3 and TIM-3 in the immune microenvironment of the tongue lesions. Representative immunohistochemically stained images of the tissue sections from the tongue. Foxp3 (**A**) and TIM-3 (**B**) staining were detected in the two groups. The expression of Foxp3 and TIM-3 in the PD-1R group were significantly higher than that in the PD-1S group, *P* < 0.05. (PDF 1750 kb)

Additional file 3: **Figure S2.** The expression of TIM-3 in Tcm and Tem cells in draining lymph nodes and spleen. (**A**) The expression of TIM-3 on CD4^+^/CD8^+^ Tem cells between PD-1R group and PD-1S groups were analyzed by flow cytometry. Representative flow cytometry dot plots show the analysis of TIM-3 expression on CD4^+^ and CD8^+^ Tcm cells(left). The expression level of TIM-3 in Tcm cells were higher in lymph nodes of PD-1R group(right). (**B**) The expression of TIM-3 on CD4^+^/CD8^+^ Tem cells between PD-1S group and PD-1R group was analyzed by flow cytometry. Representative flow cytometry dot plots show the analysis of TIM-3 expression on CD4^+^ and CD8^+^ Tem cells(left). The expression level of TIM-3 in CD8^+^ Tem cells were higher in lymph nodes of PD-1R group(right). All data represent the average ± SEM. Statistical significance was determined by Student’s t test, *P < 0.05, **P < 0.01. (PDF 1770 kb)

## Abbreviations

4-NQO: 4-nitroquinoline-1-oxide
CTLA-4: Cytotoxic T lymphocyte associated protein 4
LAG-3: Lymphocyte-activation gene 3
MDSC: myeloid derived suppressor cell
OPL: Oral precancerous lesion
PD-1: Programmed cell death 1
PD-L1: Programmed cell death ligand 1
Tcm: Central memory T cell
Tem: Effector memory T cell
TIM-3: T cell immunoglobulin and mucin domain-containing protein 3
Treg: Regulatory T cell
