# Correction to: Contributions of T cell dysfunction to the resistance against anti-PD-1 therapy in oral carcinogenesis

**DOI:** 10.1186/s13046-022-02364-8

**Published:** 2022-04-19

**Authors:** Liling Wen, Huanzi Lu, Qiusheng Li, Qunxing Li, Shuqiong Wen, Dikan Wang, Xi Wang, Juan Fang, Jun Cui, Bin Cheng, Zhi Wang

**Affiliations:** 1 Guangdong Provincial Key Laboratory of Stomatology, Guanghua School of Stomatology, Stomatological Hospital, Sun Yat-Sen University, No.56, Lingyuan West Road, Yuexiu District, Guangzhou, 510055 Guangdong People’s Republic of China; 2 Key Laboratory of Gene Engineering of the Ministry of Education, State Key Laboratory of Biocontrol, School of Life Sciences, Sun Yat-Sen University, No. 135, Xingang West Road, Haizhu District, Guangzhou, 510275 Guangdong People’s Republic of China

**Correction to: J Exp Clin Cancer Res 38, 299 (2019)**

**https://doi.org/10.1186/s13046-019-1185-0**

Following publication of the original article [1], minor errors were discovered in Figs. 1 and 4; specifically:

- Fig. 1c: an image from the PD-1R group was incorrectly used for a representative picture the Control group; the top right image has now been corrected
- Fig. 4b: the top two flow cytometry panels were duplicated in error; the top right panel has now been corrected

**Fig. 1:**
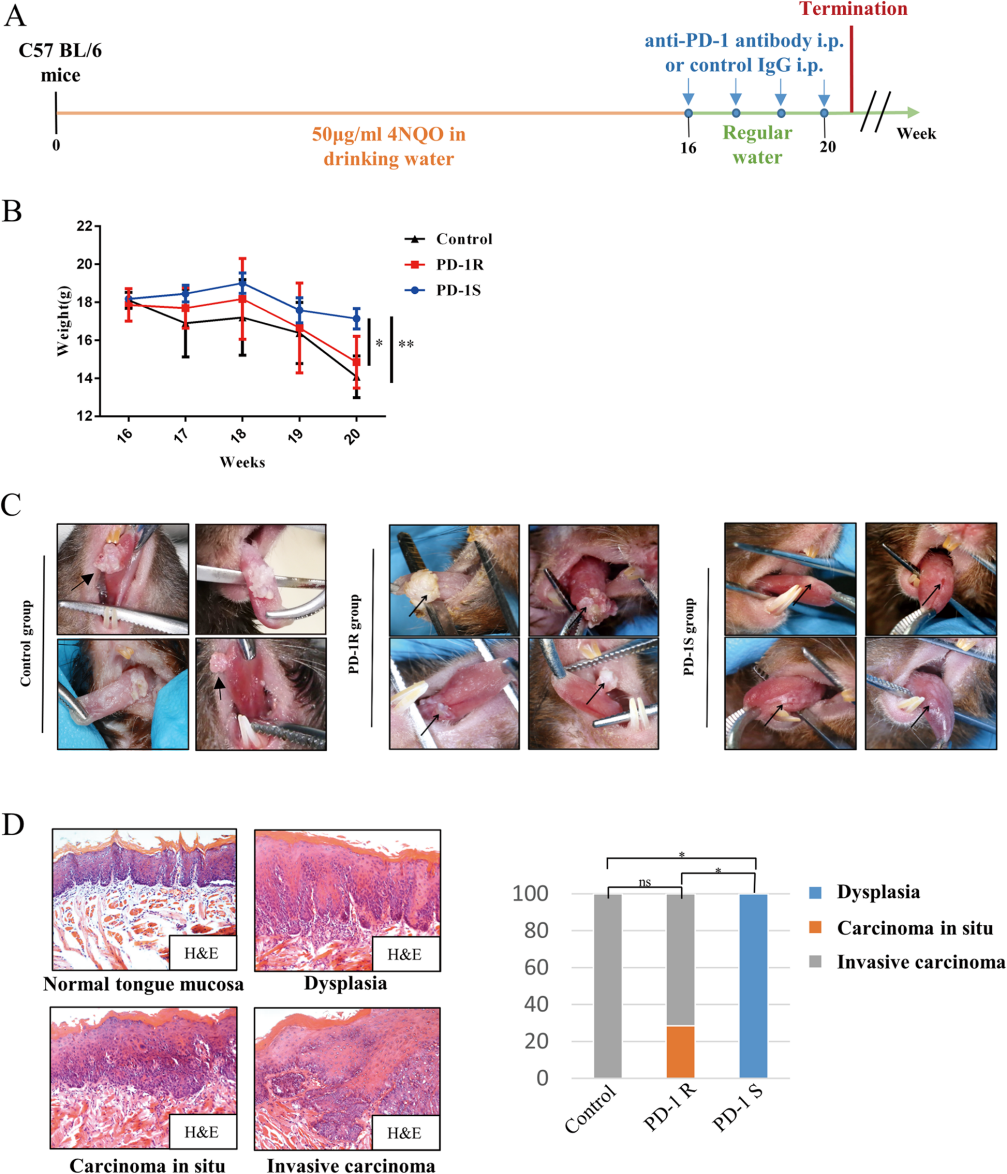
PD-1 blockade resistance occurred in the oral malignant transformation mouse model. **a** The schematic picture shows the 4NQO treatment and anti-PD-1 antibody(*n* = 23) and control IgG (vehicle control, *n* = 5) drug delivery strategies in C57BL/6 mice. **b** Body weight (g) was measured and documented for the control group and anti-PD-1 group (the PD-1R and PD-1S groups) once a week. Significant weight loss was observed in the PD-1R group at week 20. The data are presented as the mean ± SEM (one-way repeated-measures ANOVA, *P < 0.05, **P < 0.01). **c** Representative macroscopic observation of the lingual mucosal lesions after treatment with control IgG (left panel) or anti-PD-1 antibody in the PD-1R group (middle panel) and PD-1S group (right panel). For PD-1R group, similarly with control group, leukoplakia-like lesions with smooth surfaces progressed into white masses with cauliflower-like (upper left), rough and granular (upper right) or exogenous verrucous surfaces (lower right and left). The lingual mucosal lesions treated with anti-PD-1 antibodies maintained a wrinkled paper-like appearance macroscopically in PD-1S group. **d** Representative hematoxylin and eosin (H&E) staining of dysplasia, carcinoma in situ (pre-invasive carcinoma) and invasive carcinoma. Statistical significance was determined by the Kruskal-Wallis test, *P < 0.05

**Fig. 4:**
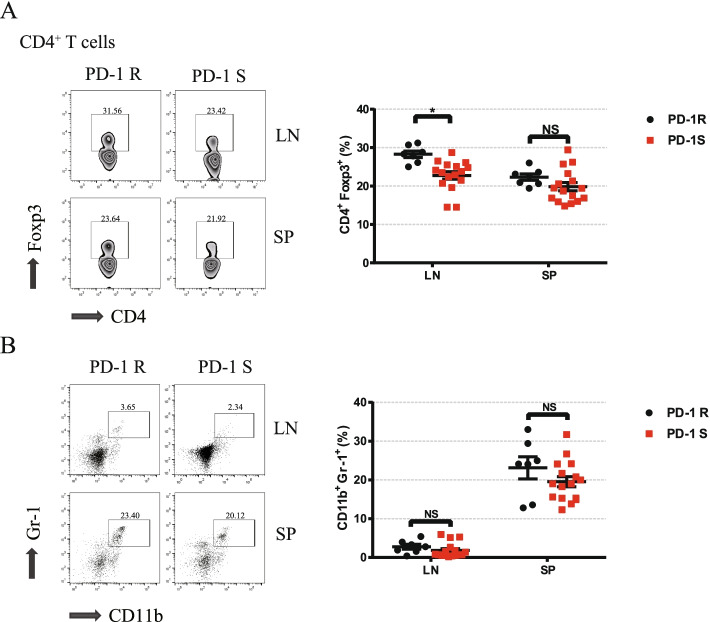
Relative distributions of key immunosuppressive cells after the anti-PD-1 antibody treatment. **a**, **b** Flow cytometry analysis was performed to characterize and quantify Tregs (CD4^+^Foxp3^+^) and MDSCs (CD11b^+^Gr-1^+^). Compared to the PD-1S group, the PD-1R group exhibited an increase in Treg accumulation. All data represent the mean ± SEM. Statistical significance was determined by Student’s t test, *P < 0.05. Tregs, regulatory T cells; MDSCs, myeloid-derived suppressor cells

The corrected figures are given here. The correction does not have any effect on the final conclusions of the paper.
