# Supplementary material for: Contributions of T cell dysfunction to the resistance against anti-PD-1 therapy in oral carcinogenesis
Source: J Exp Clin Cancer Res. 2019 Jul 10;38:299. doi: 10.1186/s13046-019-1185-0 (PMC6617956; doi:10.1186/s13046-019-1185-0)
Supplement: Supplementary file 1 — Table S1. Immunohistochemical analysis of the expression of PD-1, PD-L1 and CD3 in oral lesions. Table S2. Immunohistochemical analysis of the expression of Foxp3, TIM-3 in oral lesions. (DOCX 15 kb) [file 13046_2019_1185_MOESM1_ESM.docx]

**Additional files**

**Supplementary tables**

**Table. S1** Immunohistochemical analysis of the expression of Foxp3, TIM3 in tissue sections.

|  | **Scoring of immune stained positive**  **(Number, n(%))** | | |  |
| --- | --- | --- | --- | --- |
|  | 1 | 2 | 3 | *P-*value |
| Foxp3 staining |  |  |  | 0.044* |
| PD-1R group(n=7) | 1(14.29%) | 4(57.14%) | 2(28.57%) |  |
| PD-1S group(n=16) | 9(56.25%) | 6(37.50%) | 1(6.25%) |  |
| TIM3 staining |  |  |  | 0.022^*^ |
| PD-1R group(n=7) | 1(14.29%) | 3(42.85%) | 3(42.85%) |  |
| PD-1S group(n=16) | 11(68.75%) | 4(25.00%) | 1(6.25%) |  |

*P* value was determined by Kruskal-Wallis test. * *P* < 0.05.

**Table. S2** Immunohistochemical analysis of the expression of PD-1, PD-L1 and CD3 in tissue sections.

|  | **Scoring of immune stained positive**  **(Number, n(%))** | | |  |
| --- | --- | --- | --- | --- |
|  | **1** | **2** | **3** | ***P-*value** |
| PD-1 staining |  |  |  | 0.737 |
| control group(n=5) | 2(40.00%) | 2 (40.00%) | 1(20.00%) |  |
| PD-1R group(n=7) | 4(57.14%) | 2(28.57%) | 1(14.29%) |  |
| PD-1S group(n=16) | 9(56.25%) | 6(37.50%) | 1(6.25%) |  |
| PD-L1 staining |  |  |  | 0.022^*^ |
| control group(n=5) | 1(20.00%) | 2(40.00%) | 2(40.00%) |  |
| PD-1R group(n=7) | 1(14.29%) | 4(57.14%) | 2(28.57%) |  |
| PD-1S group(n=16) | 11(68.75%) | 4(25.00%) | 1(6.25%) |  |
| CD3 staining |  |  |  | 0.060 |
| control group(n=5) | 1(20.00%) | 2(40.00%) | 2 (40.00%) |  |
| PD-1R group(n=7) | 4(57.14%) | 2(28.57%) | 1(14.29%) |  |
| PD-1S group(n=16) | 1 (6.25%) | 7 (43.75%) | 8 (50.00%) |  |

*P* value was determined by Kruskal-Wallis test. * *P* < 0.05.
