# Supplementary material for: Contributions of T cell dysfunction to the resistance against anti-PD-1 therapy in oral carcinogenesis
Source: J Exp Clin Cancer Res. 2019 Jul 10;38:299. doi: 10.1186/s13046-019-1185-0 (PMC6617956; doi:10.1186/s13046-019-1185-0)
Supplement: Supplementary file 2 — Figure S1. Expression of Foxp3 and TIM-3 in the immune microenvironment of the tongue lesions. Representative immunohistochemically stained images of the tissue sections from the tongue. Foxp3 (A) and TIM-3 (B) staining were detected in the two groups. The expression of Foxp3 and TIM-3 in the PD-1R group were significantly higher than that in the PD-1S group, P < 0.05. (PDF 1750 kb) [file 13046_2019_1185_MOESM2_ESM.pdf]

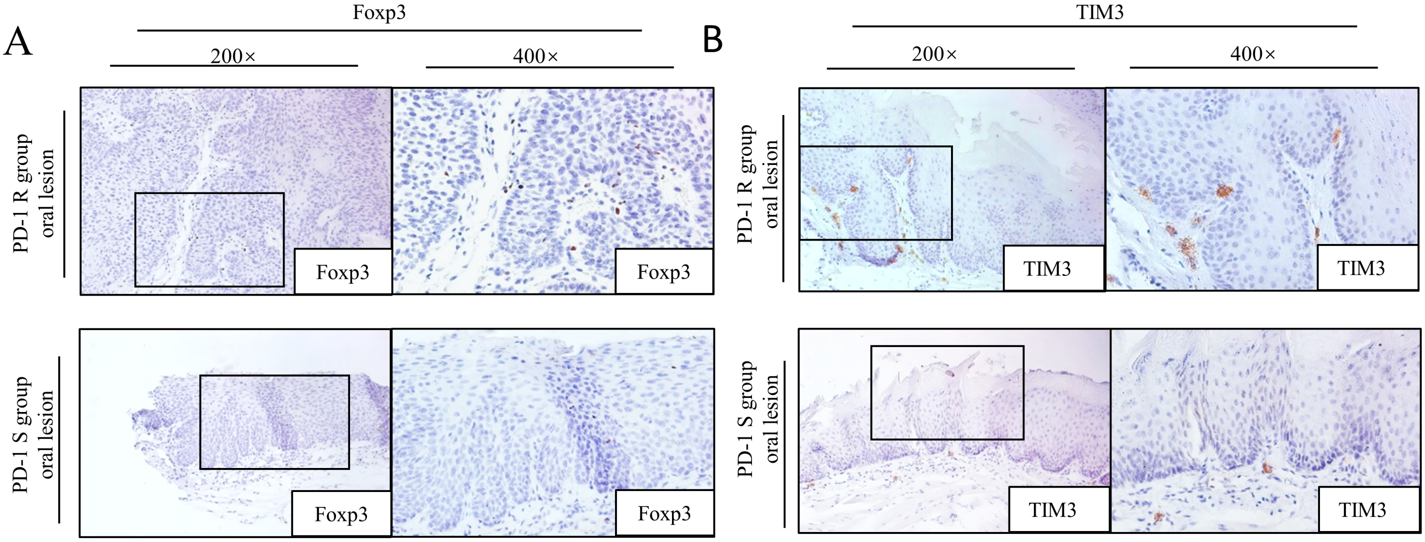

Fig. S1 Expression of Foxp3 and TIM-3 in the immune microenvironment of the tongue lesions. Representative immunohistochemically stained images of the tissue sections from the tongue. Foxp3 (A) and TIM-3 (B) staining were detected in the two groups. The expression of Foxp3 and TIM3 in the PD-1R group were significantly higher in the PD-1S group, $P < 0.05$. The positive cells in per surface were counted under 400× magnification, and five randomly selected independent microscopic fields were counted for each sample to ensure that the data were representative and homogeneous. For each biopsy, both the intensity of membrane staining (scored as + weak, ++ moderate, and +++ strong) and the percentage of positive cells was semiquantitatively evaluated for each marker in tissues according to following score: –, no; +, <10%; ++, 10–50%; +++, 51–90%, and ++++, >90% immunopositive cells, in 5 high-power (400×) fields.
