# Supplementary material for: Contributions of T cell dysfunction to the resistance against anti-PD-1 therapy in oral carcinogenesis
Source: J Exp Clin Cancer Res. 2019 Jul 10;38:299. doi: 10.1186/s13046-019-1185-0 (PMC6617956; doi:10.1186/s13046-019-1185-0)
Supplement: Supplementary file 3 — Figure S2. The expression of TIM-3 in Tcm and Tem cells in draining lymph nodes and spleen. (A) The expression of TIM-3 on CD4+/CD8+ Tem cells between PD-1R group and PD-1S groups were analyzed by flow cytometry. Representative flow cytometry dot plots show the analysis of TIM-3 expression on CD4+ and CD8+ Tcm cells(left). The expression level of TIM-3 in Tcm cells were higher in lymph nodes of PD-1R group(right). (B) The expression of TIM-3 on CD4+/CD8+ Tem cells between PD-1S group and PD-1R group was analyzed by flow cytometry. Representative flow cytometry dot plots show the analysis of TIM-3 expression on CD4+ and CD8+ Tem cells(left). The expression level of TIM-3 in CD8+ Tem cells were higher in lymph nodes of PD-1R group(right). All data represent the average ± SEM. Statistical significance was determined by Student’s t test, *P < 0.05, **P < 0.01. (PDF 1770 kb) [file 13046_2019_1185_MOESM3_ESM.pdf]

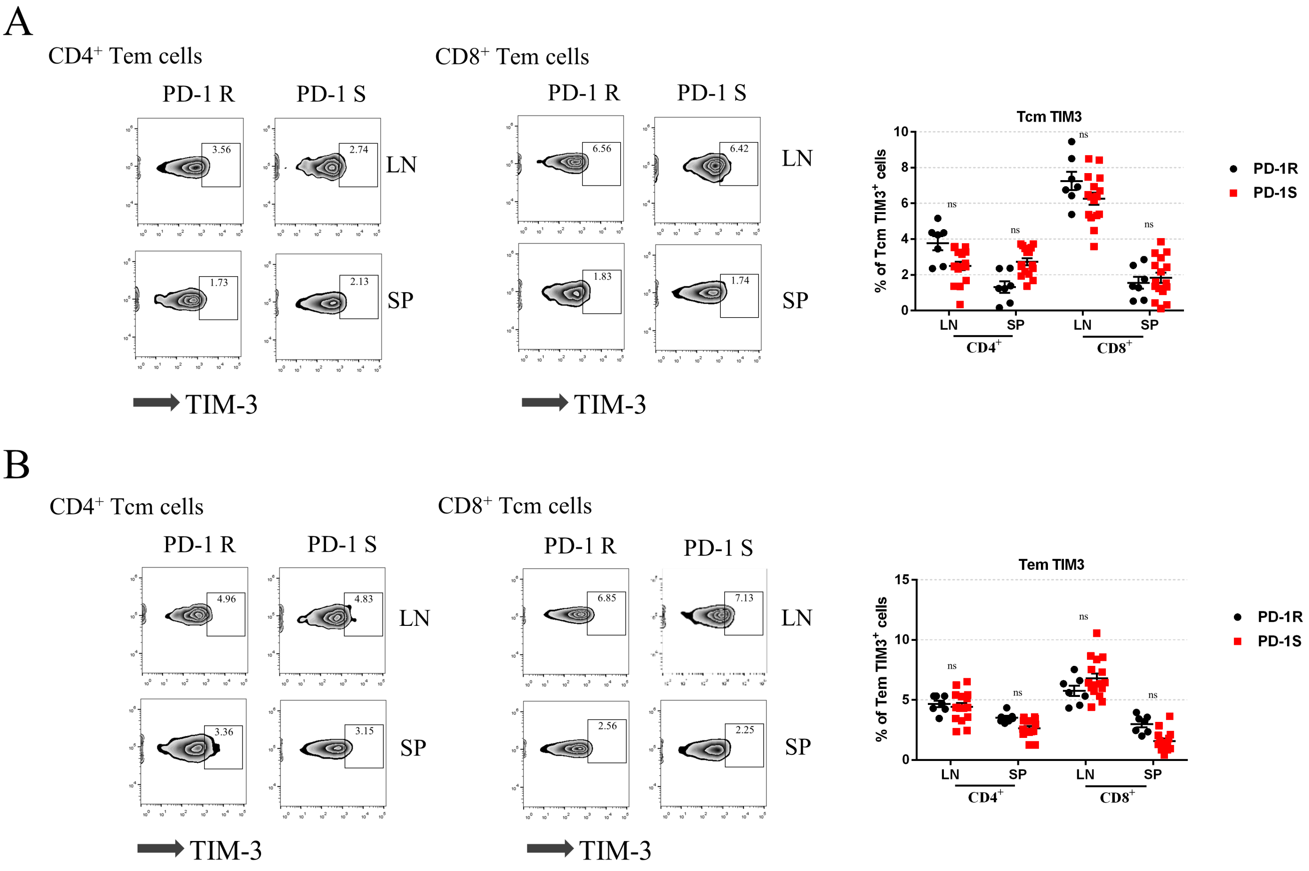

Fig. S2 The expression of TIM-3 in Tcm and Tem cells in draining lymph nodes and spleen. (A) The expression of TIM-3 on CD4+/CD8+ Tem cells between PD-1R group and PD-1S groups were analyzed by flow cytometry. Representative flow cytometry dot plots show the analysis of TIM-3 expression on CD4+ and CD8+ Tcm cells(left). The expression level of TIM-3 in Tcm cells were higher in lymph nodes of PD-1R group(right). (B) The expression of TIM-3 on CD4+/CD8+ Tem cells between PD-1S group and PD-1R group was analyzed by flow cytometry. Representative flow cytometry dot plots show the analysis of TIM-3 expression on CD4+ and CD8+ Tem cells(left). The expression level of TIM-3 in Tem cells were higher in CD8+ lymph nodes of PD-1R group(right). All data represent the average ± SEM. Statistical significance was determined by Student’s t test, *P < 0.05, **P < 0.01.
